# Spatial Organization of Osteoclastic Coupling Factors and Their Receptors at Human Bone Remodeling Sites

**DOI:** 10.3389/fmolb.2022.896841

**Published:** 2022-06-14

**Authors:** Xenia G. Borggaard, Malene H. Nielsen, Jean-Marie Delaisse, Christina M. Andreasen, Thomas L. Andersen

**Affiliations:** ^1^ Research Unit of Pathology, Department of Clinical Research and Department of Molecular Medicine, Molecular Bone Histology Team, Clinical Cell Biology, University of Southern Denmark, Odense, Denmark; ^2^ Department of Pathology, Odense University Hospital, Odense, Denmark; ^3^ Department of Forensic Medicine, Aarhus University, Aarhus, Denmark

**Keywords:** bone remodeling, osteoclast (OC), coupling, osteoblast (OB), bone cells interaction

## Abstract

The strictly regulated bone remodeling process ensures that osteoblastic bone formation is coupled to osteoclastic bone resorption. This coupling is regulated by a panel of coupling factors, including clastokines promoting the recruitment, expansion, and differentiation of osteoprogenitor cells within the eroded cavity. The osteoprogenitor cells on eroded surfaces are called reversal cells. They are intermixed with osteoclasts and become bone-forming osteoblast when reaching a critical density and maturity. Several coupling factors have been proposed in the literature, but their effects and expression pattern vary between studies depending on species and experimental setup. In this study, we investigated the mRNA levels of proposed secreted and membrane-bound coupling factors and their receptors in cortical bone remodeling events within the femur of healthy adolescent human controls using high-sensitivity RNA *in situ* hybridization. Of the proposed coupling factors, human osteoclasts showed mRNA-presence of *LIF*, *PDGFB*, *SEMA4D*, but no presence of *EFNB2*, and *OSM*. On the other hand, the osteoblastic reversal cells proximate to osteoclasts presented with *LIFR*, *PDGFRA* and *PLXNB1*, but not *PDGFRB*, which are all known receptors of the proposed coupling factors. Although *EFNB2* was not present in mature osteoclasts, the mRNA of the ligand-receptor pair *EFNB2:EPHB4* were abundant near the central blood vessels within intracortical pores with active remodeling. *EPHB4* and *SEMA4D* were also abundant in mature bone-forming osteoblasts. This study highlights that especially *LIF:LIFR*, *PDGFB:PDGFRA*, *SEMA4D:PLXNB1* may play a critical role in the osteoclast-osteoblast coupling in human remodeling events, as they are expressed within the critical cells.

## Introduction

Bone remodeling is responsible for maintenance of the adult human skeleton. Imbalances in bone resorption and formation during the bone remodeling process causes either a gain or loss of bone (Delaisse et al., 2020). Such imbalance may be due to uncoupled resorption and formation, as characteristic of ageing, osteoporosis and multiple myeloma (Andersen et al., 2010, 2013; Jensen et al., 2014; Andreasen et al., 2020). The bone remodeling process includes three different phases: First, the resorptive phase where osteoclasts start resorption of old bone. Second, the reversal-resorption phase, where osteoclasts expand the resorbed area. In the reversal-resorption phase, osteoclasts are intermixed with osteoprogenitors recruited to the eroded bone surface. These osteoprogenitors, known as reversal cells, prepare the bone surface for bone formation, while gradually undergoing differentiation into mature bone forming osteoblasts. Third, the bone formation phase (Andersen et al., 2013; Lassen et al., 2017; Delaisse et al., 2020; Sims and Martin, 2020).

The proximity between osteoclasts and osteoblastic reversal cells during the reversal-resorption phase allows active interaction and signaling between these cell types. Furthermore, reversal cells comprise a possible target cell available for osteoclastic coupling factors. Osteoclastic coupling factors include: 1) matrix-derived factors released during resorption, 2) factors secreted by osteoclasts, 3) membrane-bound factors on osteoclasts, and 4) factors packed in exosomes released by osteoclasts (Charles and Aliprantis, 2014; Sims and Martin, 2020).

The discovery of reversal cells vacating eroded bone surfaces near osteoclasts have provided a potential bridge in the communication between osteoclasts and osteoblastic cells during bone remodeling, as bone-resorbing osteoclasts are rarely observed near bone-forming osteoblasts (Eriksen, Melsen and Mosekilde, 1984; Andersen et al., 2009; Lassen et al., 2017). Initially, reversal cells were described as mononucleated cells, covering approximately 80% of eroded surfaces in trabecular bone and proposed to be pre-osteoclasts due to the presence of TRAcP (Baron et al., 1983; Eriksen et al., 1984; Eriksen et al., 1984; Bianco et al., 1988; Mocetti et al., 2000). We now know that they are osteoblast-lineage cells, expressing markers specific for early osteogenic commitment (Andersen et al., 2009; Abdelgawad et al., 2016; Abdallah et al., 2017; Jafari et al., 2017; Lassen et al., 2017; Chen et al., 2019). Furthermore, we have shown that early reversal cells have direct cell-cell interactions with osteoclasts, they take up Tartrate-resistant acid phosphatase (TRAcP) released by osteoclasts, and they decompose resorption debris left by the osteoclast (Everts et al., 2002; Abdelgawad et al., 2016). Collectively, this supports the concept that osteoblastic reversal cells are a key recipient of osteoclastic coupling factors (Charles and Aliprantis, 2014; Delaisse et al., 2020).

The proposed osteoclastic coupling factors include secreted and membrane-bound coupling factors. Potential secreted coupling factors (clastokines) includes Leukemia Inhibitory Factor (LIF), Cardiotrophin-1 (CTF1) and Oncostatin M (OSM) from the IL-6 family of cytokines. These cytokines have been suggested to play a role in bone metabolism (Sims, 2009, 2021). An interesting feature of these cytokines is their dependency of the glycoprotein 130 subunit during signaling, and their ability to react with other receptors within this group of cytokines (Kishimoto et al., 1995). LIF has been associated with metabolic and immunological processes and especially with growth and bone metabolism (Ware et al., 1995; Jones and Jenkins, 2018). The receptor of LIF (LIFR) is expressed by murine osteoblastic cells *in vitro* (Allan et al., 1990; Reid et al., 1990; Bellido et al., 1997; Walker et al., 2010). The amino acid sequence of CTF1 is similar to LIF and able to bind and activate LIFR(Pennica et al., 1995). In primary murine osteoblastic cells, *Ctf1* expression increases with differentiation (Liu, Aubin and Malaval, 2002) whereas CTF1 protein has been reported in mature murine osteoclasts (Walker et al., 2008). OSM also has the ability to bind and activate LIFR (Rose and Bruce, 1991; Liu et al., 1992) besides the specific OSM receptor (OSMR) (Thoma et al., 1994). Murine osteoblastic cells express both *Lifr* and *Osmr*, but their expression levels differ throughout differentiation (Bellido et al., 1996). However, knowledge on how *LIF*, *CTF1*, *OSM*, *OSMR* and *LIFR* are expressed in human bone is scarce.

Platelet-Derived Growth Factor (PDGF) has also attracted attention as a possible secreted coupling factor regulating bone formation (Horner et al., 1996). PDGFs are dimeric proteins of two polypeptide chains, forming either homodimers (AA, BB) or heterodimers (AB). Likewise, PDGF receptors are dimeric and either homodimers or heterodimers (PDGFRA, PDGFRB or PDGFRAB). PDGF-BB is considered the universal PDGF with binding affinity for all PDGF receptors (Horner et al., 1996; Alvarez, Kantarjian and Cortes, 2006). In human trabecular bone, *PDGFB* expression was recently observed in osteoclasts, while its receptors *PDGFRA* and *PDGFRB* were expressed in proximate reversal cells and osteoblastic canopy cells, separating bone surface cells from the marrow cavity (Brun et al., 2020).

Proposed membrane-bound coupling factors include semaphorin 4D (SEMA4D), a transmembrane glycoprotein with high affinity to PlexinB1 (PLXNB1) (Kang and Kumanogoh, 2013). SEMA4D is believed to be a repressor of bone formation, as knockdown in mice leads to a high bone-mass phenotype with no effect on bone resorption (Negishi-Koga et al., 2011). In humans, high serum levels of SEMA4D has been associated with low BMD and decreased markers of bone formation (Zhang et al., 2015). However, not much is known about the spatial expression of *SEMA4D* and *PLXNB1* within the bone environment. EphrinB2 (EFNB2) is yet another proposed membrane-bound coupling factor. EFNB2 is a transmembrane ligand of the receptor tyrosine kinase EPHB4. Activation of receptor tyrosine kinases initiates bidirectional signaling, forward through the receptor and reverse through the ligand (Pasquale, 2010; Taylor, Campbell and Nobes, 2017). Expression of *Efnb2* has been shown in osteoclasts, osteoblasts and osteocytes of mice, whereas *Ephb4* expression has only been shown in osteoblasts (Arthur et al., 2011, 2018; Wang et al., 2014).

In this study we investigated spatial mRNA localization of several suggested coupling factors, secreted or membrane-bound in osteoclasts, and their receptors in osteoblastic reversal cells and osteoblasts within human intracortical bone remodeling events.

## Materials and Methods

Human bone specimens were collected from the proximal femur of nine adolescent patients aged 6–15 years undergoing corrective surgery for Coxa Valga. Collected specimens were fixated in 4% paraformaldehyde for 2 days and subsequently decalcified for 30 days in 0.5 M EDTA containing 0.4% paraformaldehyde. Decalcified specimens were dehydrated, paraffin-embedded and cut in series of 3.5-µm-thick adjacent sections. Every fifth section was Masson Trichrome stained to select samples with active bone remodeling (identified as erosion or formation in cortical pores). Selected sections were stained with *in situ* hybridization combined with TRAcP. Spatial localization of each mRNA was validated in at least three different individuals. The study was approved by the Danish National Committee on Biomedical Research Ethics (Project-ID: S-2012-0193).

### *In situ* Hybridization Combined With Immunostaining

Sections adjacent to Masson Trichrome stained sections were *in situ* hybridized for the mRNA abundance of proposed coupling factors *LIF*, *CTF1*, *OSM*, *SEMA4D*, *EPHB4* and *PDGFA*, as well as their receptors *LIFR*, *OSMR*, *PLXNB1*, *EFNB2*, *PDGFRA* and *PDGFRB*. *In situ* hybridization was performed using a modified RNAscope 2.5 high-definition procedure (R2283, Sigma-Aldrich). After deparaffinization and rehydration, sections were treated with 1.5% hydrogen peroxidase for 30 min at room temperature to inactivate endogenous peroxidases. Subsequently, sections were pretreated with RNAscope Target Retrieval for 15 min at 90°C and pepsin (322300, ACD Bioscience) for 20 min at 40°C. After pretreatment, sections were hybridized in a HybEZ™ hybridization oven at 40°C overnight with 20- probe-pairs for human *LIF* (cat. No: 445721, binding nt 839-1780 of NM_002309.4), *CTF1* (Cat. No. 895601, binding nt 40-1222 of NM_001330.5), *OSM* (Cat. No. 456381, binding nt 32-1175 of NM_020530.4), *SEMA4D* (Cat. No, 430711, binding nt 611-1623 of NM_006378.3), EPH receptor panel with high affinity for *EPHB4* and affinity for *EPHB1/EPHB2/EPHB3* (Cat. No. 516401, binding nt 2019-2577 of NM_004444.4), *PDGFB* (Cat. No. 406701, binding nt 665-2037 of NM_033016.2), *LIFR* (Cat. No. 441021, binding nt 2411-3421 of NM_001127671.1), *OSMR* (Cat. No. 537121, binding nt 307-1357 of NM_001323505.1), *PLXNB1* (Cat. No. 430681, binding nt 1208-2101 of NM_002673.5), *EFNB2* (Cat. No. 430651, binding nt 2-919 of NM_004093.3), *PDGFRA* (Cat. No. 604481, binding nt 844-1774 of NM_006206.4) and *PDGFRB* (Cat. No. 548991, binding 523-2984 of NM_002609.3) from ACD Bioscience. The probes were diluted 1:1 in probe diluent (449819, ACD Bioscience) and negative controls were with only probe diluent. Each probe was validated on a tissue array with 36 different anonymized tissue-samples. Hybridized probes were branch amplified through six steps in the HybEZ™ hybridization oven according to manufactures instructions, and further enhanced with digoxigenin-conjugated tyramide (NEL748001KT, PerkinElmer) detected with alkaline-phosphatase conjugated sheep anti-digoxigenin Fab fragments (11093274910, Roche) and visualized using Liquid Permanent Red (Agiliant). After the *in situ* procedure, osteoclasts were immunostained with mouse-anti-TRAcP IgG2B antibody (clone 9C5, MABF96, Merck Millipore) detected with horseradish peroxidase-conjugated anti-mouse IgG polymers (BrightVision, Immunologic, Duiven, Holland) and visualized using Deep Space Black (Biocare Medical Concord, CA, United States). Finally, sections were counterstained with Mayer’s hematoxylin.

### Microscopy

The stained sections were imaged on a VS200 slide scanner (Olympus) using Z-stack condensed into a single plane with optimal focus, which were investigated using the Olivia software (Olympus).

## Results

In this observational study, we examined the presence of mRNA encoding secreted and membrane-bound coupling factors proposed in the literature and their receptors in human cortical bone remodeling events. Here, we focused particularly on reversal cells situated adjacent to mature bone resorbing osteoclasts. We examined femur cortical bone specimens from nine adolescents and each mRNA was evaluated in at least three different individuals.

### IL-6 Family Cytokines and Their Receptors are Present in Human Bone Remodeling Events

**FIGURE 1:**
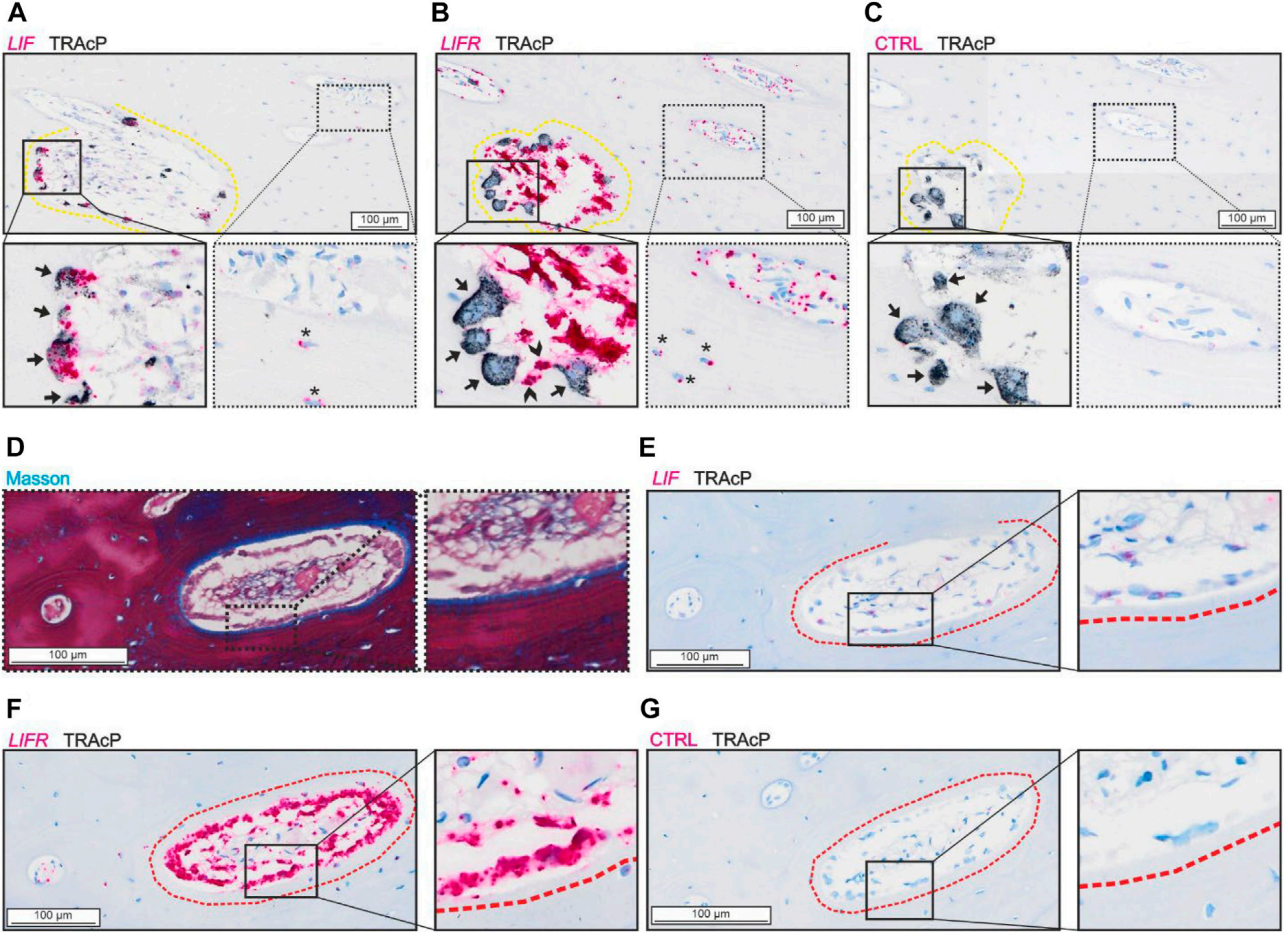
LIF is present in osteoclasts and LIFR in osteoblasts and mononucleated cells in the lumen of adjacent sections of intracortical canals. **(A–C)** Adjacent sections of eroded pore with *in situ* hybridization (red) of LIF **(A)**, LIFR **(B)** and negative control **(C)** combined with immunostaining of TRAcP (black). **(D–G)** Adjacent sections of formative pore with masson **(D)** and *in situ* hybridization of LIF **(E)**, LIFR **(F)** or negative control **(G)** combined with immunostaining of TRAcP (black). Eroded surfaces are marked by a yellow dashed line, formative bone surfaces are marked by a red dashed line. Osteoclasts are indicated by black arrows in the zooms, Reversal cells are indicated by arrowheads and osteocytes with signal from *in situ* hybridization are shown with*.

Analysis of the spatial mRNA localization of *LIF* and *LIFR* revealed a high abundance of *LIF* in mature bone-resorbing osteoclasts and a lower presence in osteocytes (Figure 1A). On the other hand, the *LIFR* mRNA was not detected in mature bone-resorbing osteoclasts (Figure 1B). Instead, *LIFR* was highly abundant in reversal cells near osteoclasts on the eroded surfaces, and in mononucleated cells within the pore lumen, which to a great extend reflect osteoprogenitors being recruited to the eroded surfaces as reversal cells (Lassen et al., 2017) (Figure 1B). *LIFR* was also abundant in mature bone-forming osteoblasts on osteoid surfaces and only weakly present in some osteocytes (Figures 1D,F). *LIF* was only weakly present in mature bone-forming osteoblasts (Figure 1E).

**FIGURE 2:**
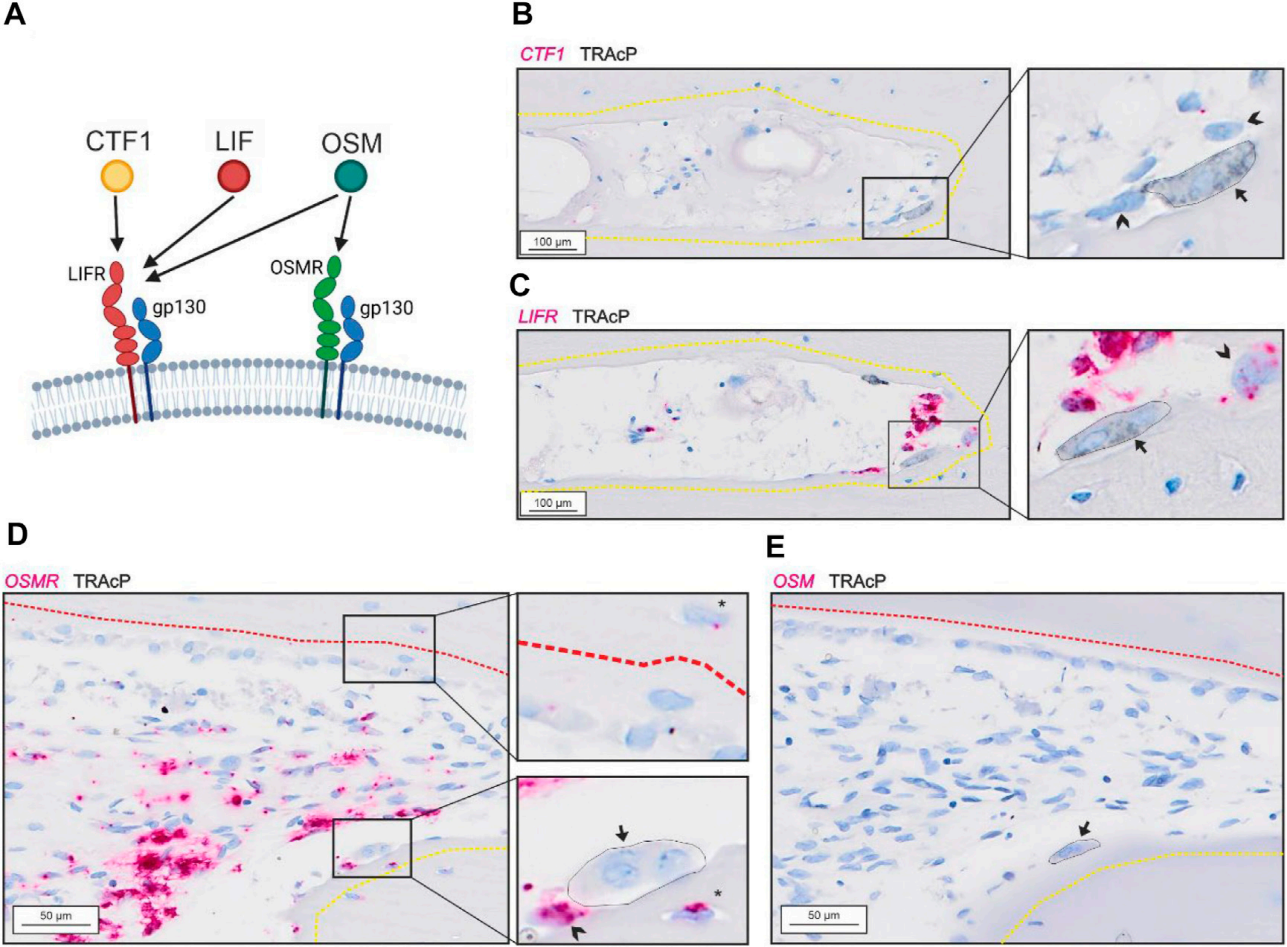
Gp130-associating receptors are present within intracortical pores with active bone remodeling. **(A)** schematic illustration of the two gp130-associating receptors (LIFR and OSMR) and their affinity for CTF-1, LIF and OSM. **(A–B)** Adjacent sections of eroded pore with *in situ* hybridization (red) of *CTF1* **(A)** and *LIFR* **(B)** combined with immunostaining of TRAcP (black). **(D–E)** Adjacent sections of pore with resorption and formation with *in situ* hybridization (red) of *OSMR* **(D)** and *OSM* **(E)** combined with immunostaining of TRAcP (black). Eroded surfaces are marked by a yellow dashed line and formative surfaces are marked by a red dashed line. Osteoclasts are marked by a black arrow and outlined in the zooms, reversal cells are marked by arrowheads.

Bone-resorbing osteoclasts showed no presence of *CTF1* (Figure 2B) despite presence of *LIFR* in proximate reversal cells and mononucleated cells within the lumen (potential osteoprogenitors) (Figure 2C). *OSMR* was abundant in reversal cells and proximate mononucleated cells within the lumen (potential osteoprogenitors), as well as to some extend in osteocytes. In contrast to *LIFR*, *OSMR* was not notably present in bone-forming osteoblasts (Figure 2D). Surprisingly, bone-resorbing osteoclasts showed no evidence of *OSM* mRNA (Figure 2E), as the case for *CTF1*. Levels of *OSM* and *CFT1* was generally low and restricted to a few mononucleated cells within the intracortical pores. Both *OSM* and *CTF1* were detected in different tissues in the control tissue array (Suppl. 1).

### PDGF and its Receptors are Present in Human Bone Remodeling Events

**FIGURE 3:**
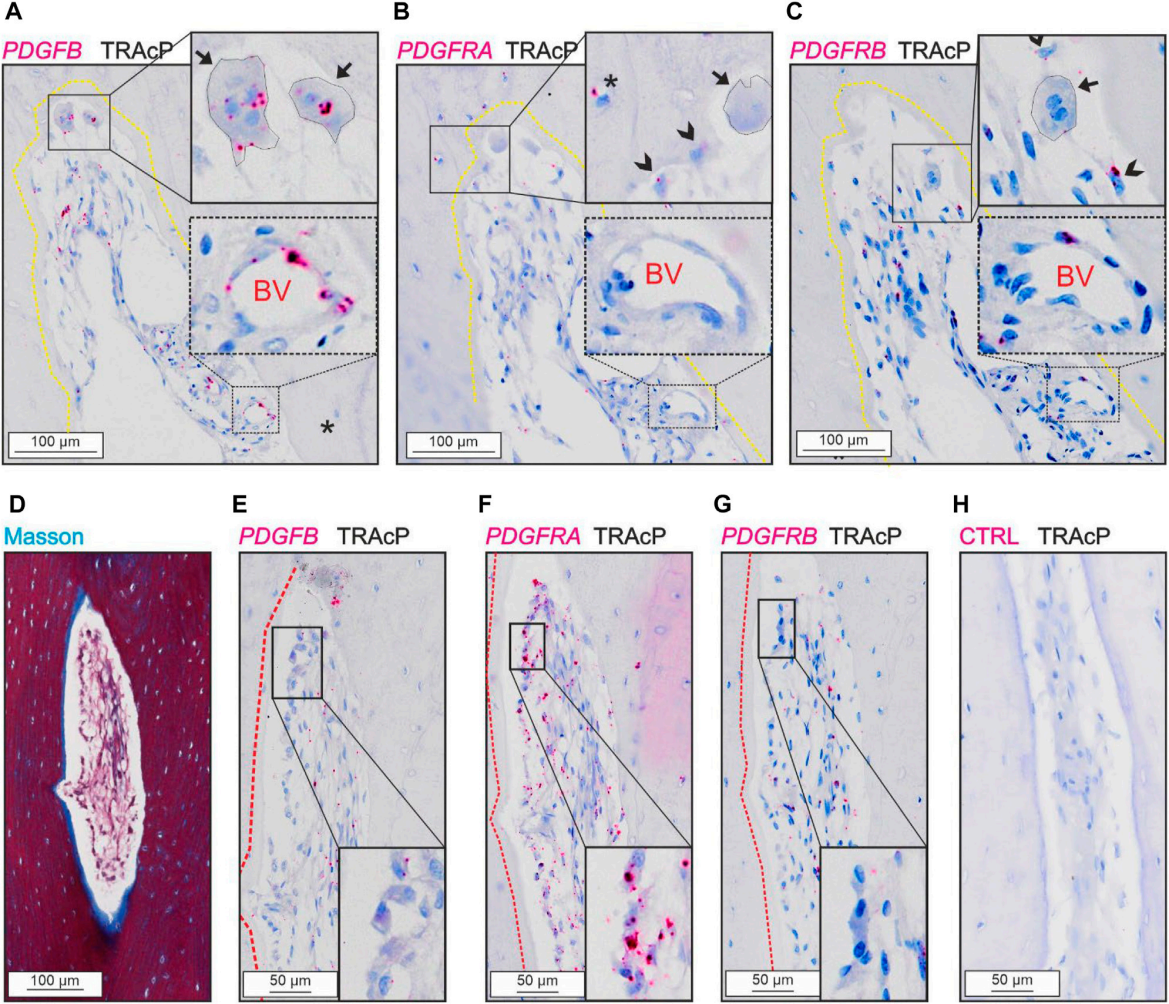
Levels of *PDGFRA* and *PDGFRB* differ in cortical bone. **(A–C)** Adjacent sections of eroded pore with immunostaining of TRAcP (black) and *in situ* hybridization (red) of *PDGFB* **(A)**, *PDGFRA* **(B)** and *PDGFRB* **(C)**. **(D–H)** Adjacent sections of pore with bone formation with Masson Trichrome **(D)** or *in situ* hybridization of *PDGFB* **(E)**, *PDGFRA* **(F)**, *PDGFRB* **(G)** or negative control **(H)** combined with immunostaining of TRAcP (black). Eroded surfaces are marked by a yellow dotted line, formative surfaces are marked by a red dotted line. Osteoclasts are outlined and marked with black arrows, reversal cells with arrowheads and osteocytes with *in situ* signal are marked with (*), blood vessels are indicated with “BV”.

*PDGFB* was detected in osteoclasts and in cells near the vascular structures, not in reversal cells (Figures 3A,E). The two receptors were present at different levels in the tissue.

*PDGFRA* and *PDGFRB* were present in reversal cells on eroded surfaces (Figures 3B, C). Furthermore, *PDGFRA* was present in osteocytes and osteoblasts (Figures 3B,F), whereas *PDGFRB* was primarily located near vascular structures within the lumen of intracortical pores and not in bone forming osteoblasts or osteocytes (Figures 3C,G).

### SEMA4D and PLXNB1 are Present in Human Bone Remodeling Events

**FIGURE 4:**
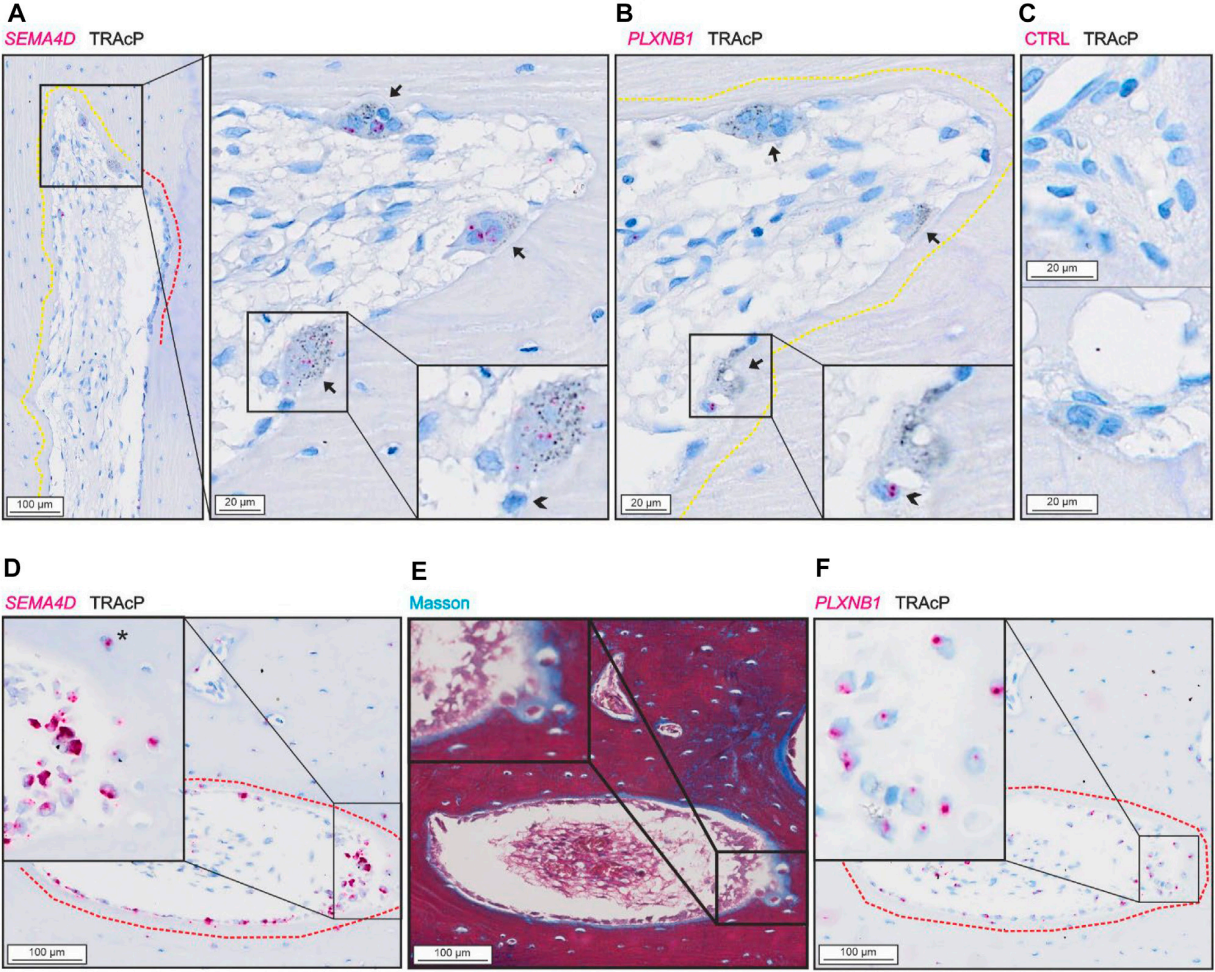
*SEMA4D* is present in osteoclasts and mature osteoblasts and *PLXNB1* is present in osteoblasts. **(A–C)** Adjacent sections of pore with resorption with *in situ* hybridization (red) of *SEMA4D* **(A)**, *PLXNB1* **(B)** or negative control **(C)** combined with immunostaining of TRAcP (black). **(D–F)** Adjacent sections of pore with bone formation with masson trichrome staining **(E)** or *in situ* hybridization (red) of *SEMA4D* **(D)** or *PLXNB1* **(F)** combined with immunostaining of TRAcP (black). Eroded surfaces are marked by a yellow dotted line, formative surfaces are marked by a red dotted line. Osteoclasts are outlined and marked with black arrows, reversal cells with arrowheads (*).

*SEMA4D* was present in mature bone-resorbing osteoclasts (Figure 4A) and in bone-forming osteoblasts on osteoid surfaces and in some osteocytes (Figures 4D,E). The few mononucleated cells within the lumen showing low levels of *SEMA4D*, appeared morphologically like endothelial cells instead of potential osteoprogenitors. Discrete levels of *PLXNB1* (receptor of SEMA4D) were observed in reversal cells next to *SEMA4D*-positive osteoclasts (Figure 4B), and in bone-forming osteoblasts on osteoid surfaces and in some osteocytes (Figures 4E,F). No *PLXNB1* was observed in mature bone-resorbing osteoclasts (Figure 4B).

### *Vascular Structures Express EFNB2 and EPHB4* in *Human Bone Remodeling Events*

**FIGURE 5:**
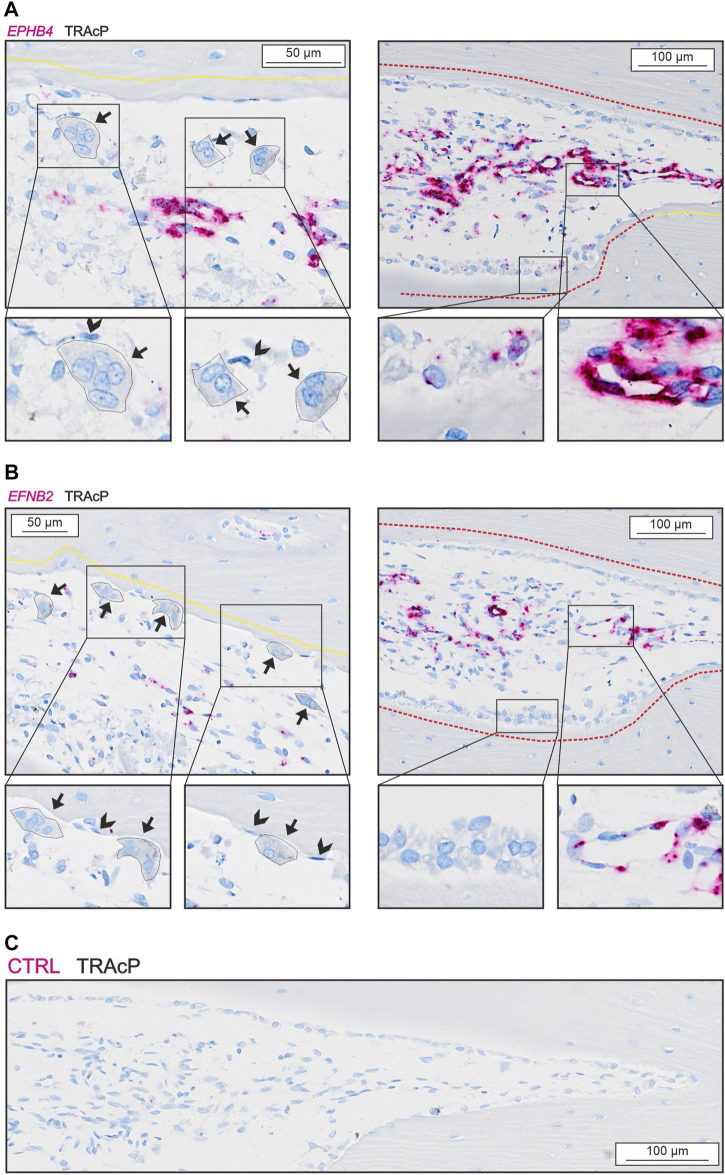
*EFNB2* and *EPHB4* are mainly present near vascular structures. **(A)** *In situ* hybridization of *EPHB4* (red) and immunohistochemical staining of TRAcP (black). **(B)** *In situ* hybridization of *EFNB2* (red) and immunohistochemical staining of TRAcP (black). Eroded surfaces are marked by a yellow dotted line, formative surfaces are marked by a red dotted line. Osteoclasts are outlined and marked with black arrows, reversal cells with arrowheads.

We observed no presence of either *EPHB4* or *EFNB2* in osteoclasts, reversal cells or osteocytes (Figure 5), but some mature bone-forming osteoblasts contained *EPHB4* mRNA (Figure 5A). In contrast, both EPHB4 and EFNB2 were highly present in vascular structures within the intracortical pores (Figure 5).

## Discussion

The elusive coupling of bone formation to osteoclastic bone resorption is a critical step in the bone remodeling process, which we are only starting to understand (Delaisse et al., 2020). Osteoclastic coupling factors play a central role in the osteoclast-osteoblast coupling, ensuring the initiation of bone formation within the vacated resorption cavities (Sims and Martin, 2020). The present study examines *in situ* mRNA localization of potential membrane-bound and secreted osteoclastic coupling factors and their respective receptors in human cortical remodeling events. The study demonstrates that mRNA of several coupling factors are present in osteoclasts, while their receptors were present in neighboring osteoblastic reversal cells (e.g., osteoprogenitors) during the reversal-resorption phase. This supports the notion that interactions between osteoclasts and osteoprogenitors within the reversal-resorption phase play a key role in the coupling mechanism, potentially involving a dedicated panel of secreted and membrane-bound coupling factors.

### Secreted Osteoclastic Coupling Factors and Their Receptors in Human Bone Remodeling

In human cortical remodeling events, the osteoclastic levels of *LIF* and high levels of *LIFR* in neighboring reversal cells and potential osteoprogenitors within the lumen, support that LIF:LIFR signaling may likely have a functional role in the coupling during human bone remodeling. This supports previous studies in genetic mice models, suggesting a pro-osteogenic effect of LIF:LIFR signaling during bone remodeling. These mice studies showed an increased bone volume when *Lif* was overexpressed (Metcalf and Gearing, 1989), and decreased bone volume and increased number of osteoclasts in *Lif* knockout mice (Bozec et al., 2008) and *Lifr* knockout mice (Ware et al., 1995). This skeletal effect is partly transferable to humans, where mutations in the *LIFR* gene cause Stüve-Wiedemann syndrome (OMIM #610559), characterized by bowing and thickening in the lower limbs and abnormal trabecular bone structure (Cormier-Daire et al., 1998; Dagoneau et al., 2004). Stüve-Wiedemann syndrome is often fatal and associated with early lethality, as also observed in *Lif* and *Lifr* knockout mice (Cormier-Daire et al., 1998; Sims, 2009). The early lethality makes the effects of LIF:LIFR signaling on remodeling versus modeling and growth hard to interpret, and differing roles of LIF signaling in bone development and remodeling has been reported (Poulton et al., 2012). Our findings of *LIF* and *LIFR* in interacting osteoclasts and osteoprogenitors, support that LIF:LIFR signaling plays a role in the osteoclast-osteoblast coupling within the reversal-resorption phase.

Importantly, LIFR signaling can also be activated by several other ligands of the IL-6 family cytokines (Kishimoto et al., 1995). CTF1 and OSM are two alternative ligands of LIFR, which have been suggested to play a regulatory role in bone remodeling. Like LIF, CTF1 might possess different roles in modeling versus remodeling events. Studies on *Ctf1* knockout mice have shown that they are osteopenic at birth but had a high bone mass phenotype at 10- and 26-weeks of age (Walker et al., 2008; Poulton et al., 2012). In the same study, CTF1 protein was observed in murine osteoclasts. We did not observe any notable levels of *CTF1* mRNA in osteoclasts, reversal cells or osteoblasts in human cortical bone remodeling events, questioning its importance in human bone remodeling. OSM is an alternative ligand of LIFR, which has been extensively studied. Studies treating mice with OSM has indicated both pro-osteogenic effects (Jay et al., 1996; Bellido et al., 1997; Walker et al., 2010), as well as an increased osteoclast formation and activity (Tamura et al., 1993; Palmqvist et al., 2002). Recently, it was suggested that OSM signaling through LIFR stimulates bone formation (Walker et al., 2010), consistent with a high bone mass phenotype observed in mice overexpressing bovine *Osm* (Malik et al., 1995). Conversely, OSM signaling through OSMR is suggested to induce osteoclastogenesis indirectly by upregulating RANKL expression (Walker et al., 2010). However, we did not observe any notable presence in osteoclasts, reversal cells or osteoblasts in human cortical bone remodeling events, questioning its importance in human bone remodeling. On the other hand, we did observe *OSMR* mRNA in reversal cells and potential osteoprogenitors within the lumen of intracortical pores, which may respond to an alternative unknown ligand.

Another potential secreted osteoclastic coupling factor is PDGF homodimers or heterodimers, which have attracted attention as regulators of bone remodeling. This attention originates from clinical studies with the tyrosine kinase inhibitors Imatinib and Nilutinib observed to increase serum markers of bone formation, but not resorption (Grey et al., 2006). Subsequently, *in vitro* studies ascribed this effect of Imatinib and Nilutinib treatment to PDGFR-β signaling causing increased *Opg* expression (O’Sullivan et al., 2007, 2011, 2016). Treatment with PDGF-BB has also been shown to increase mesenchymal cell proliferation and osteoblast differentiation *in vitro*, but also the expression of pro-resorptive factors, such as *Csf1* and *Rankl* (Chen et al., 2015). In human trabecular bone, *PDGFB* was expressed by osteoclasts whereas both PDGF receptors (*PDGFRA* and *PDGFRB*) were expressed by osteoblastic canopy cells and reversal cells (Brun et al., 2020). In the present study, we observed expression of both receptors in reversal cells but differing expression pattern in other cells. Besides in reversal cells, *PDGFRA* was expressed by osteocytes and osteoblasts, whereas *PDGFRB* was expressed near vascular structures within intracortical pores.

### Membrane-Bound Osteoclastic Coupling Factor and Their Receptors in Human Bone Remodeling

In human cortical bone remodeling events, *SEMA4D* was present in osteoclasts and *PLXNB1* was observed in reversal cells, supporting that SEMA4D:PLXNB1 binding may play a role in their communication. This is in line with murine studies, showing *Sema4d* expression in osteoclasts and osteoclast progenitors, and increased *Plxnb1* expression during osteoblast differentiation (Negishi-Koga et al., 2011). Functional studies in mice, suggest that Sema4D is a suppressor of bone formation with knockdown leading to a higher bone mass. However, the cause of high bone mass in knockout mice does not concur between studies. Negishi-Koga and colleagues reported increased bone formation without osteoclastic effect (Negishi-Koga et al., 2011) whereas Dacquin and colleagues observed reduced resorptive activity (Dacquin et al., 2011). In a clinical study, serum levels of SEMA4D positively correlated with serum markers of resorption in patients with multiple myeloma (Zhang et al., 2015; Terpos et al., 2018). Later, SEMA4D secreted from a human lung cancer cell line were shown to inhibit osteoblast differentiation *in vitro* (Chen et al., 2019). In contrast to this study, treatment of osteoporotic postmenopausal women with the antiresorptive Denosumab have been shown to increase serum levels of SEMA4D compared to controls (Anastasilakis et al., 2015), suggesting that SEMA4D is produced by other sources than osteoclasts. This study suggests that SEM4D originate from mature bone-forming osteoblasts, showing presence of *SEMA4D* mRNA at human bone remodeling sites.

EFNB2:EPHB4 signaling has also been proposed as a coupling pathway requiring cell-cell contact. *Efnb2* and *Ephb4* have been reported in several bone cells (Arthur et al., 2011, 2018; Wang et al., 2014) and EFNB2:EPHB4 signaling within the osteoblast lineage is believed to promote osteoblast differentiation (Takyar et al., 2013; Tonna et al., 2014). Nevertheless, we were unable to observe any notable presence of *EFNB2* and *EPHB4* in human osteoclasts and reversal cells questioning its direct importance in the osteoclast-osteoblasts coupling mechanism of human bone remodeling. On the other hand, *EFNB2* and *EPHB4* are highly expressed in the vascular structures within the lumen of intracortical pores, consistent with a role in the local vascularization and angiogenesis as shown in other studies (Wang et al., 2010). Vascularization is essential for osteoprogenitor recruitment and thereby indirectly the activation of bone formation on eroded bone surfaces vacated by the osteoclasts.

In this study, we qualitatively investigated the spatial *in situ* mRNA localization of proposed coupling factors and their receptors using bone specimens from adolescents undergoing corrective surgery for Coxa Valga. Therefore, we consider the analyzed cortical bone as healthy. By investigating intracortical pores, we ensure that well-defined remodeling processes were examined, despite the young age of patients. Our investigations are limited to the *in situ* cellular mRNA-levels, which are affected by expression and stability of each individual mRNA. Despite the use of a tissue array to validate probes, stability and retention time within bone may vary from other tissues. The study does not investigate the distribution of proteins or functional analyses of included coupling factors. In the applied mRNA detection-procedure we used probe pairs designed by ACD Bioscience. Each set of probe pairs included 20 different probe pairs targeting a specific region within the gene of interest. Levels of mRNA detected were described af high/low when compared to other probes or differing levels between cell types.

Further investigation of the mRNA and protein abundance, as well as functional significance of these coupling factors are needed in human bone remodeling.

## Conclusion

**FIGURE 6:**
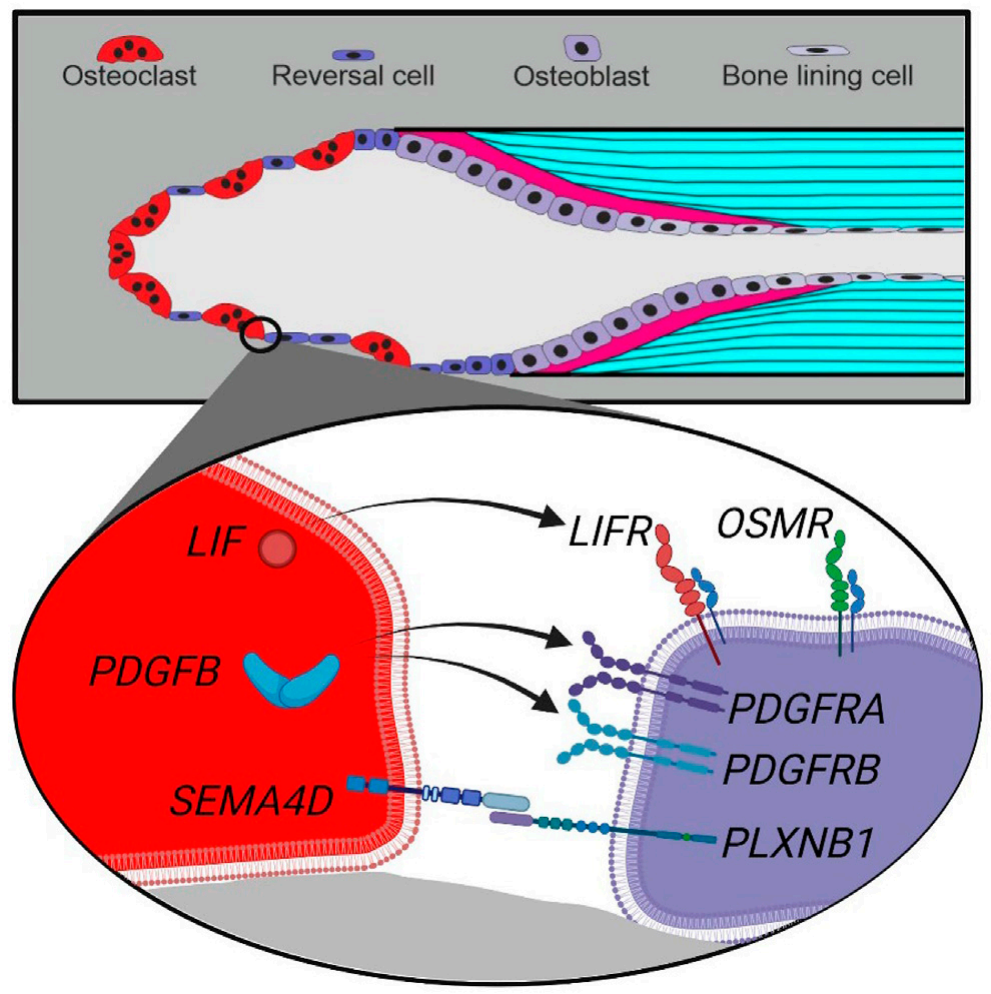
Schematic presentation of a cutting cone with observed coupling factors and receptors in osteoclasts and reversal cells in human bone remodeling. Top, a cutting cone with initial resorption (left), reversal-resorption phase with osteoclasts intermixed with reversal cells (middle) followed by bone forming osteoblasts laying down osteoid (right). Bottom, zoom on the cell-cell interface between osteoclasts (red) and reversal cells (purple) with observed expression of coupling factors and receptors.

Our mRNA analysis of human cortical bone remodeling events revealed presence of proposed coupling factors *LIF*, *SEMA4D* and *PDGFB* mRNA in mature bone-resorbing osteoclasts and presence of their respective receptors *LIFR*, *PLXNB1*, *PDGFRA* and *PDGFRB* mRNA in neighboring reversal cells. These results are complementary to previous functional studies, supporting a functional role in the coupling mechanism of human bone remodeling. Conversely, we did not observe presence of *CTF1* or *OSM* mRNA in mature osteoclasts, despite the presence of *OSMR* mRNA in neighboring reversal cells (Figure 6). Finally, presence of *EFNB2* and *EPHB4* mRNA was restricted to vascular structures within intracortical pores, with no indications of presence within osteoclasts nor reversal cells.

## Data Availability

The original contributions presented in the study are included in the article/Supplementary Material, further inquiries can be directed to the corresponding author.
